# Case Report: Ocular metastasis from ALK-rearranged pulmonary adenocarcinoma presenting as a pseudo-syndrome of anterior uveitis

**DOI:** 10.3389/fonc.2025.1619667

**Published:** 2025-08-13

**Authors:** Cong Zhao, Jiaqi Chen, Hongji Liu, Yan Dai

**Affiliations:** ^1^ EyeSchool of Chengdu University of Traditional Chinese Medicine, Chengdu, Sichuan, China; ^2^ Department of Ophthalmology, Mianyang Central Hospital, School of Medicine, University of Electronic Science and Technology of China, Mianyang, Sichuan, China

**Keywords:** metastasis, non-small cell lung cancer, ALK mutation, alectinib, case report

## Abstract

Lung cancer is the second most common primary site for intraocular metastatic tumors, with the most frequent metastatic site being the choroid. However, cases of intraocular metastasis of lung cancer presenting as anterior uveitis or secondary glaucoma are rare and often misdiagnosed. Here, we report a case of a lung adenocarcinoma stage IV patient, who presented with anterior uveitis as the initial symptom without respiratory symptoms. After 28 months of follow-up, the patient received targeted treatment with Alectinib hydrochloride capsules and a series of timely ophthalmic surgeries. Following these treatments, the patient’s intraocular and intracranial metastatic lesions disappeared, the primary pulmonary lesion significantly shrank, and the best corrected visual acuity (BCVA) improved from HM/30cm to 1.0. No significant toxic side effects were observed during the treatment, and the prognosis was favorable. The patient is currently living and working normally without complications. This case highlights the importance of considering metastatic tumors in patients with refractory anterior uveitis. Combined multimodal imaging and fluid biopsy can improve the early diagnosis rate of intraocular metastases. Targeted therapy based on genetic mutation detection, along with the appropriate timing for ophthalmic surgery, is crucial for improving patient prognosis.

## 1 Introduction

Lung cancer is the second most common primary site for intraocular metastatic tumors, accounting for 39% to 49% of all intraocular malignancies. The majority of these ocular metastases are located in the choroid (88%), with a small portion found in the iris (9%) and ciliary body (2%) (1–3). The clinical manifestations are varied, including iris masses, inflammation of the iris and ciliary body, and secondary glaucoma (4). This poses significant challenges for the early accurate identification of such rare cases and the development of appropriate treatment strategies (5). Comprehensive ocular and cranial examination are required, and diagnosis can be confirmed through histological or cytological analysis. Furthermore, imaging modalities such as magnetic resonance imaging (MRI) and positron emission tomography/computed tomography (PET/CT) are non-invasive techniques that do not exert mechanical disruption to tissues, thereby minimizing the potential risk of tumor cell dissemination or seeding. As such, they serve as effective alternatives or complementary tools to invasive diagnostic procedures (6, 7).

Human tumor genetic mutation testing is an important tool for guiding cancer targeted therapy (8). The anaplastic lymphoma kinase (ALK) fusion is one of the key driver genes in non-small cell lung cancer (NSCLC) (9). ALK-tyrosine kinase inhibitors (ALK-TKIs) have demonstrated significant therapeutic effects for ALK-positive NSCLC patients (10, 11). However, reports on targeted therapy for iris and ciliary body metastases based on ALK gene testing in NSCLC are rare. This case report discusses a patient with lung adenocarcinoma who initially presented with right eye anterior uveitis as a symptom and was treated with the ALK inhibitor Alectinib hydrochloride capsules. This study aims to provide insights and valuable information for the management of NSCLC with iris and ciliary body metastatic tumors.

## 2 Case description

**Figure 1:**
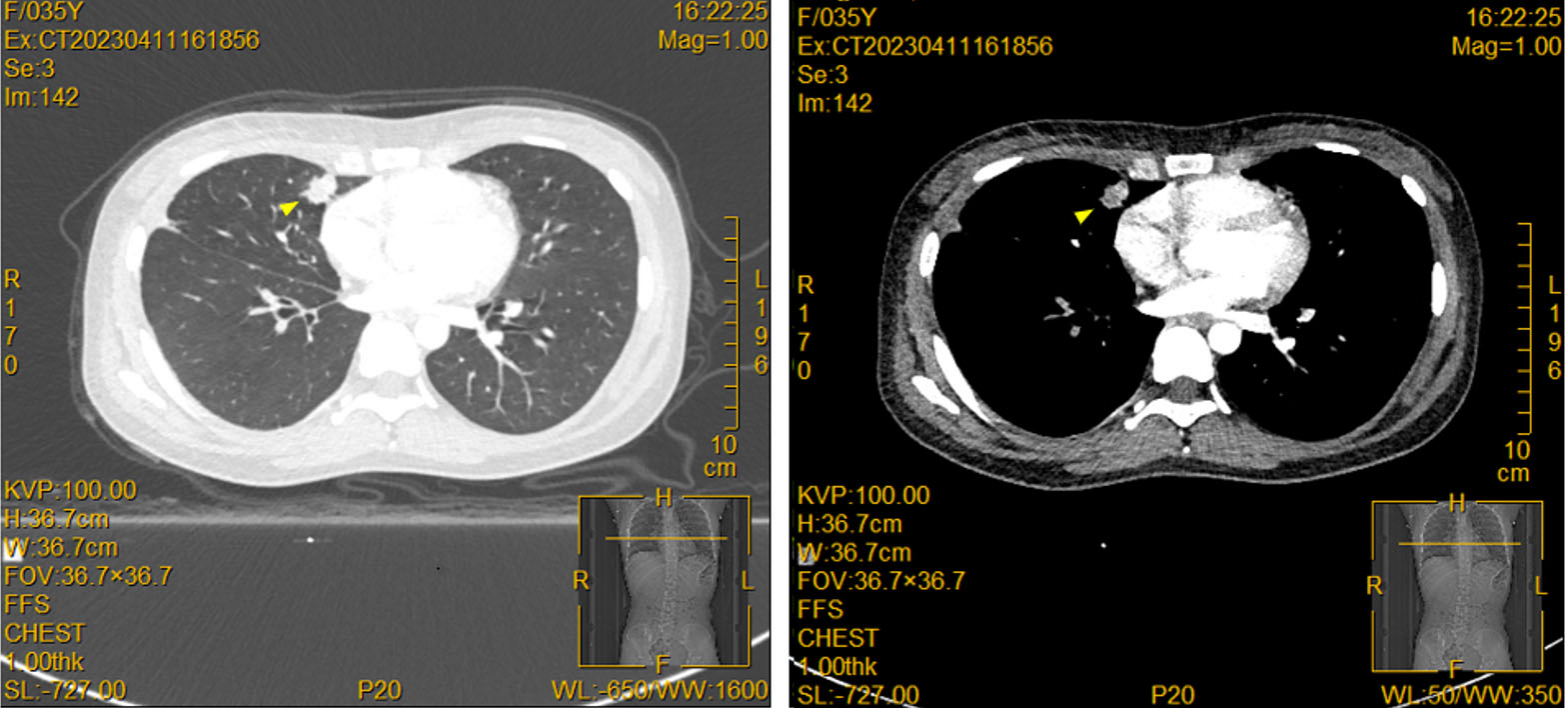
Pulmonary lesions are displayed in the contrast-enhanced CT. A solid nodule (yellow triangle) was observed in the inner segment of the right middle lobe (Img120), measuring approximately 16.6mm × 12.1mm, with lobulated and spiculated edges, and showing heterogeneous enhancement on contrast imaging.

**Figure 2:**
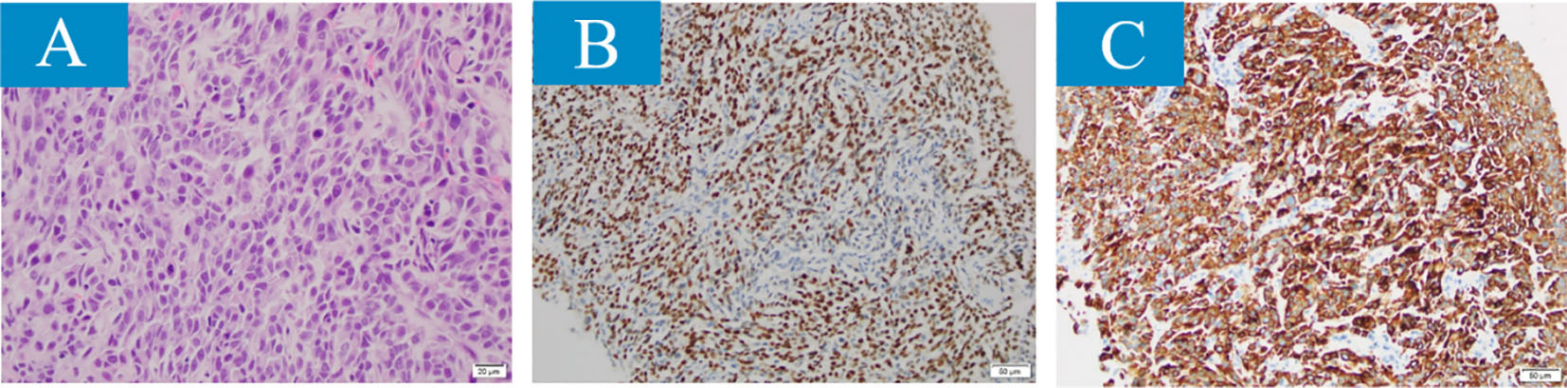
The histopathological results revealed features of small cell lung cancer in the lesion samples obtained through fiberoptic bronchoscopy (magnification × 400): **(A)** poorly differentiated, scattered darkly stained nuclear heterogeneous cell clusters, tumor cells arranged in solid sheet-like patterns, with visible atypia, increased nucleocytoplasmic ratio, and evident mitosis. **(B, C)** Immunohistochemical characteristics. **(B)** Tumor cells positive for cytokeratin, **(C)** positive for thyroid transcription factor-1 (TTF-1).

The patient is a 36-year-old woman who had a 28-month history of right eye anterior uveitis and secondary glaucoma for two months. She also had a history of right eye vision loss, eye redness, and eye pain, but no other discomforts and no history of systemic diseases. Ocular examination revealed a BCVA of HM/30cm in the right eye. The intraocular pressure was 43 mmHg in the right eye. There was significant anterior segment inflammation in the right eye, with corneal edema, keratic precipitates (KP++), anterior chamber reaction (+), cells (++), with a shallowing of the anterior chamber. The pupil was irregular, dilated about 5 mm, with no light reflex, and the iris was fully adhered posteriorly. The iris texture was unclear, with numerous grayish-white nodules on the surface. The lens was partially cloudy, and the fundus was unclear (**Figure 1**). Pupillary block and obstruction of the aqueous humor outflow pathway were identified as the primary causes of elevated intraocular pressure in this patient. However, treatment with anti-inflammatory agents, mydriatics, and intraocular pressure-lowering drugs, the effect was unsatisfactory. A chest X-ray showed a nodule in the right hilar region, and further chest CT revealed a nodule in the right middle lobe (inner segment), measuring approximately 16.6 mm ×12.1 mm, with lobulated, spiculated edges (**Figure 1**). To clarify the nature of the lesion, a biopsy was performed through bronchoscopy, and pathological samples were obtained from the lung lesion. Histologically, the lung specimen showed scattered atypical cell clusters with a hemorrhagic background (**Figure 2**). Immunohistochemical staining showed positive expression of P-CK, CK7, TTF1, CD44V6, and Ki-67, with negative expression of P40 and CK5/6. The diagnosis was stage IV lung adenocarcinoma.

**Figure 3:**
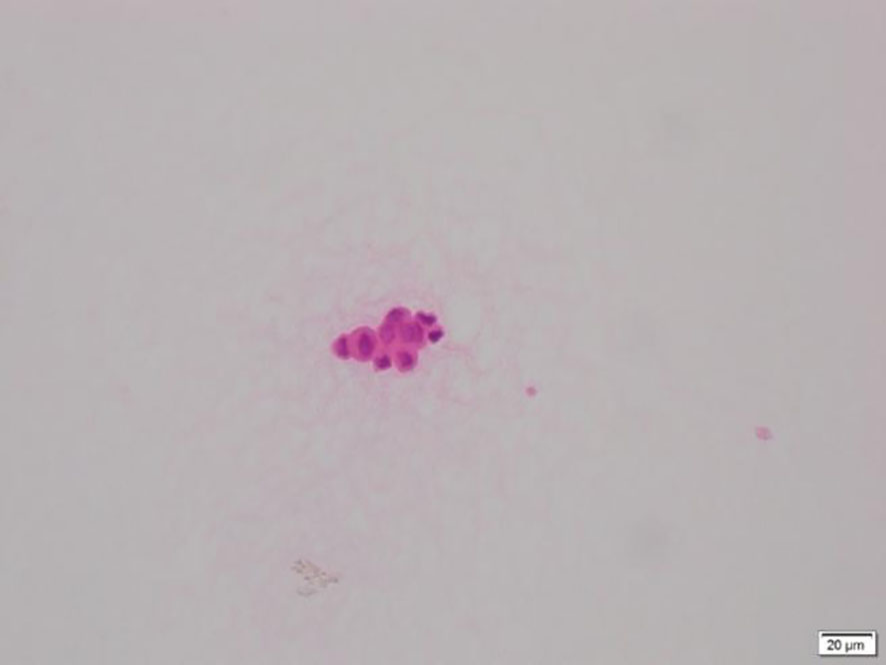
Histological features of aqueous humor cells stained with Hematoxylin and Eosin (H&E): Small clusters of atypical cells are observed, with an increased nucleocytoplasmic ratio, and both the cells and their nuclei show atypia.

**Figure 4:**
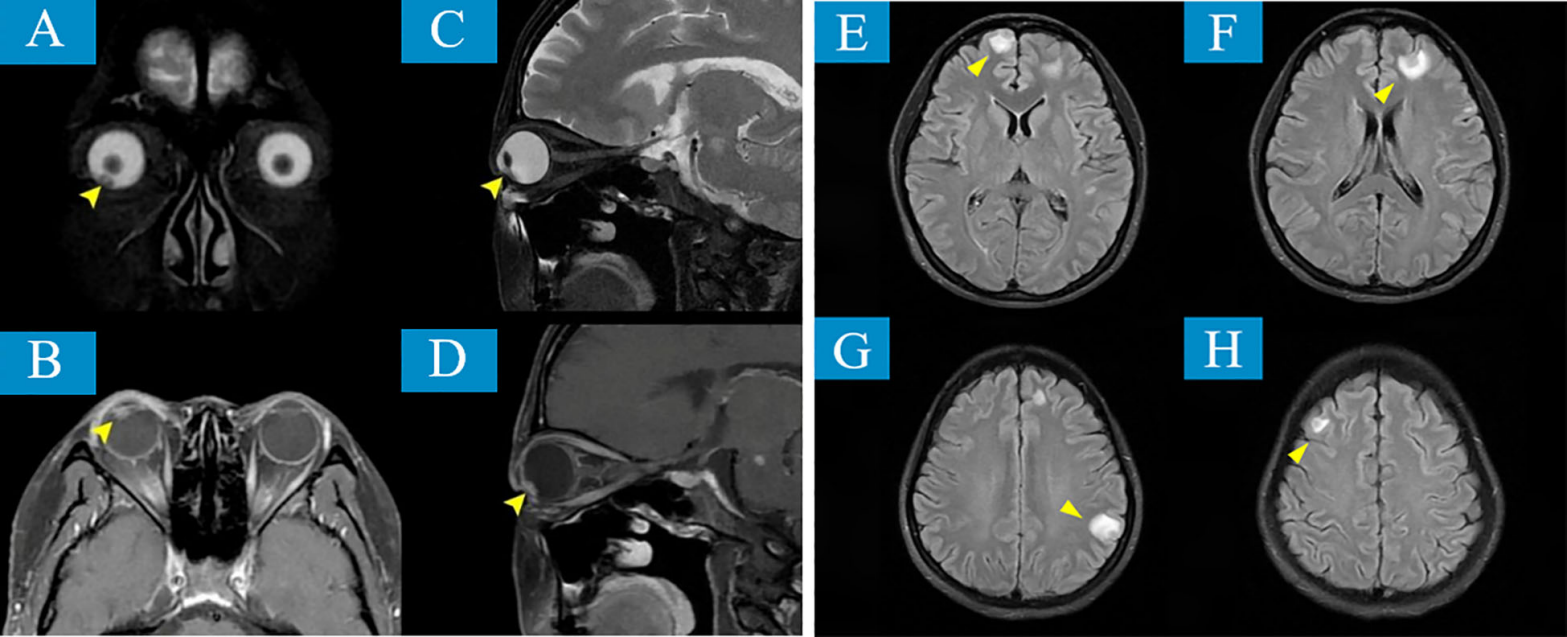
MRI reveals lesions involving the right eyeball and intracranial regions. **(A, B)** T2-weighted sequences (T2WI) and **(C, D)** T1-weighted sequences (T1WI) reveal an isointense mass, whose location is identified as the inferotemporal iris and ciliary body of the right eye based on ophthalmic anatomy (yellow arrows). In addition, multiple nodular abnormal signal intensities are observed in the left pons, bilateral cerebellar hemispheres, frontal and parietal lobes, and the left temporal lobe (yellow arrows) **(E–H)**.

Given the rarity of ocular metastasis from lung cancer, an aqueous humor smear from the right eye was performed (**Figure 3**), revealing a small number of tumor cells in the smear, with small atypical cell clusters. The nuclear-to-cytoplasm ratio was increased, and both the cells and their nuclei showed atypical features. Cranial MRI also confirmed (**Figure 4**) a nodular lesion on the lower anterior wall of the right eyeball, with isointensity on T1-weighted imaging and slightly increased signal intensity on T2-weighted imaging. Based on ophthalmic anatomy, the lesion was identified to be located in the inferotemporal iris and ciliary body of the right eye. Additionally, multiple nodular abnormal signals were observed in the left pons, bilateral cerebellar hemispheres, frontal parietal lobes, and left temporal lobe. The signal intensity was slightly low on T1-weighted imaging and slightly increased on T2-weighted imaging. These findings were consistent with ocular and intracranial metastases from lung cancer.

**Figure 5:**
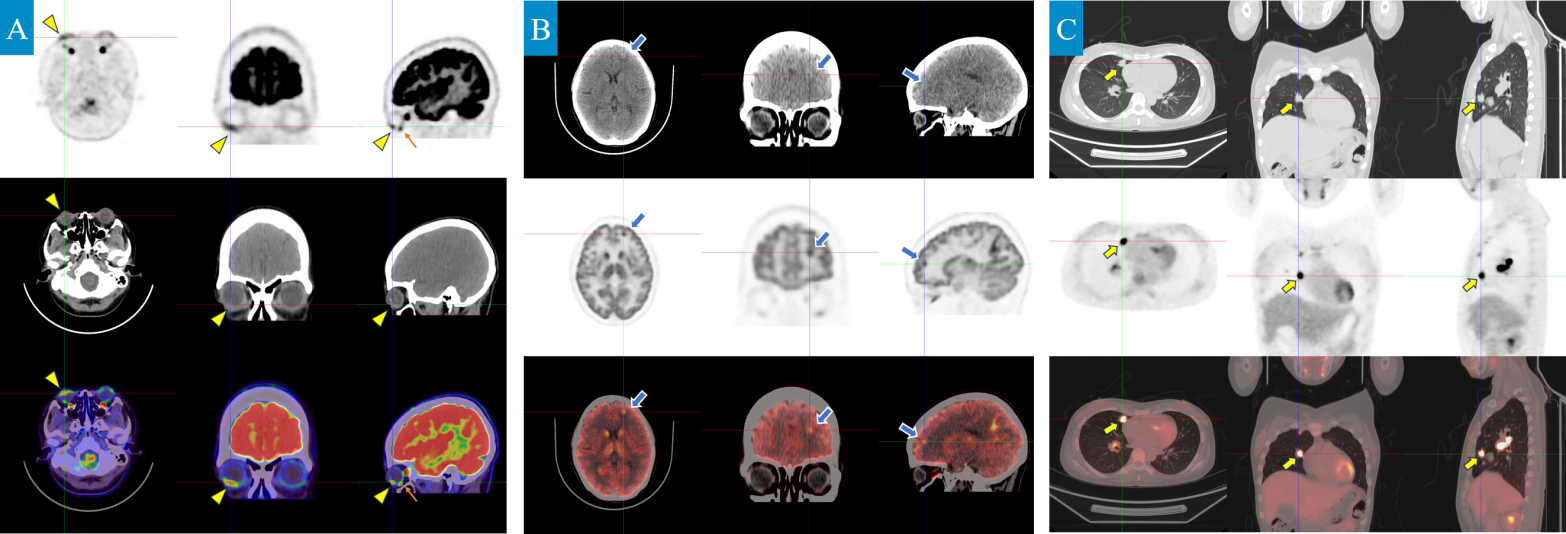
PET/CT scan shows metastatic lesions and the primary lung lesion (axial, coronal, and sagittal planes). **(A)** A slightly hyperdense nodule is observed in the anterior-inferior wall of the right eyeball, slightly toward the right (yellow triangle), with ill-defined margins and increased glucose metabolism, showing a maximum standardized uptake value (SUVmax) of approximately 4.6. (Note: The orange arrow indicates an area of high metabolic uptake in the extraocular muscles. No corresponding abnormal signal was detected on MRI, confirming that this is physiological uptake by muscle tissue and is unrelated to metastatic lesions.) **(B)** A ring-shaped high-density nodule, approximately 8mm × 8mm in size, was observed in the left frontal lobe, with patchy slightly lower-density areas around it (blue arrow). The contrast agent uptake was increased, with an SUVmax of approximately 12.3 **(C)**. In the right middle lobe, a solid nodule approximately 16mm × 12mm in size was found in the medial segment, with lobulated edges and spiculated features of varying lengths (yellow arrow). The contrast agent uptake was increased, with an SUVmax of approximately 11.1. Another nodule, measuring less than 0.6cm in diameter, was found in the right middle lobe, with short spicules on part of the edges. Increased contrast agent uptake was noted, with an SUVmax of approximately 4.4. Scattered nodular lesions, with a diameter of no more than 0.5cm, were seen in the right lung, with clear borders and slightly increased contrast agent uptake, showing an SUVmax of approximately 1.0.

To rule out other possible lesions, a positron emission tomography/computed tomography (PET/CT) scan was performed. The results confirmed intracranial and ocular metastases: a slightly higher-density nodule was observed in the right lower anterior wall of the eye, with ill-defined borders and increased glucose metabolism, with an SUVmax of approximately 4.6; a ring-shaped high-density nodule was found in the left frontal lobe, measuring about 8mm × 8mm, with an SUVmax of approximately 12.3. Additionally, small lymph nodes were noted to be enlarged in the right axillary tail region, with an SUVmax of about 2.0 (**Figure 5**).

**Figure 6:**
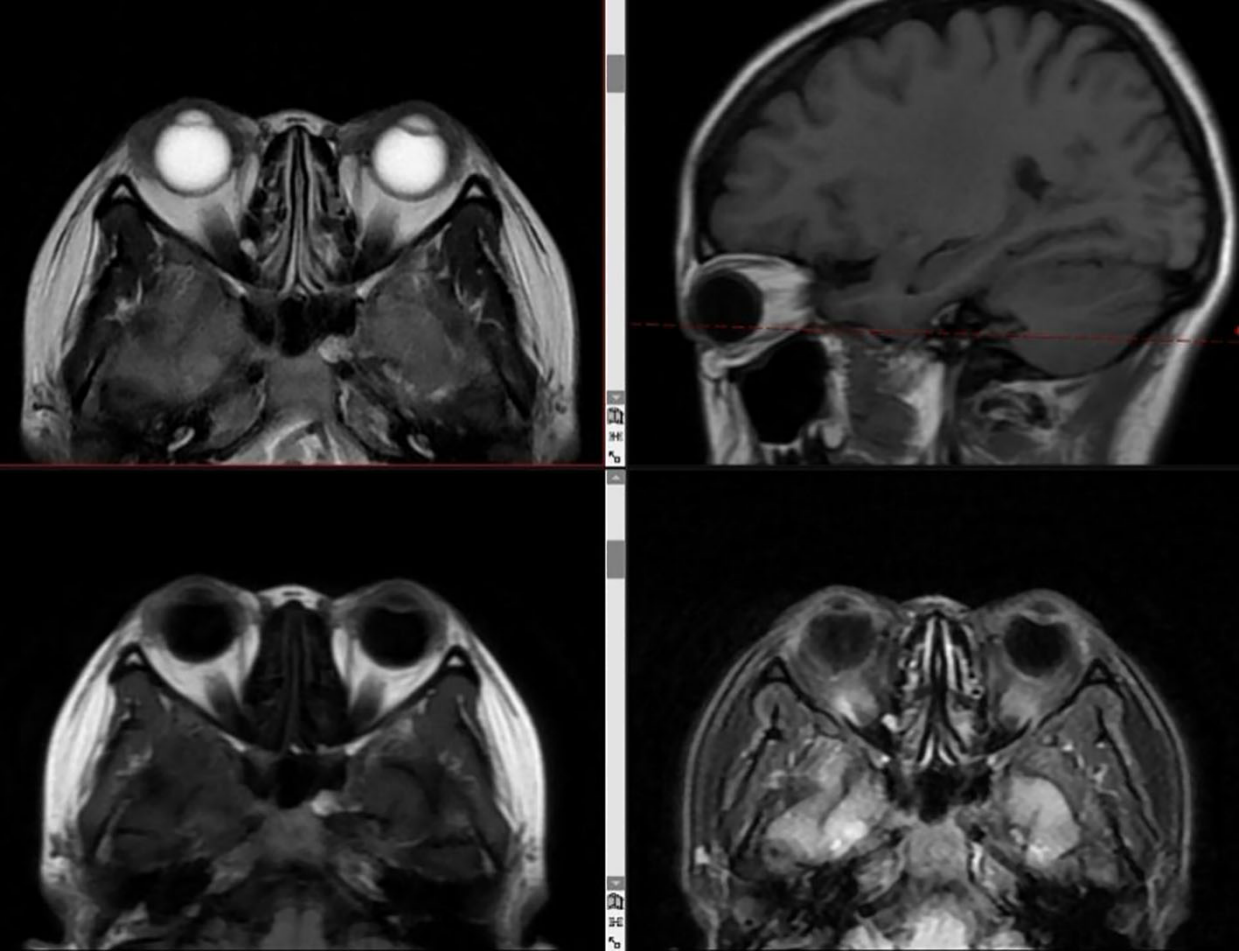
MRI shows complete resolution of ocular and intracranial lesions after treatment. T1WI, T2WI, T2Flair, and DWI images show no abnormal signals in the eye region; no abnormal enhancement was observed after contrast administration. All brain structures appear normal.

**Figure 7:**
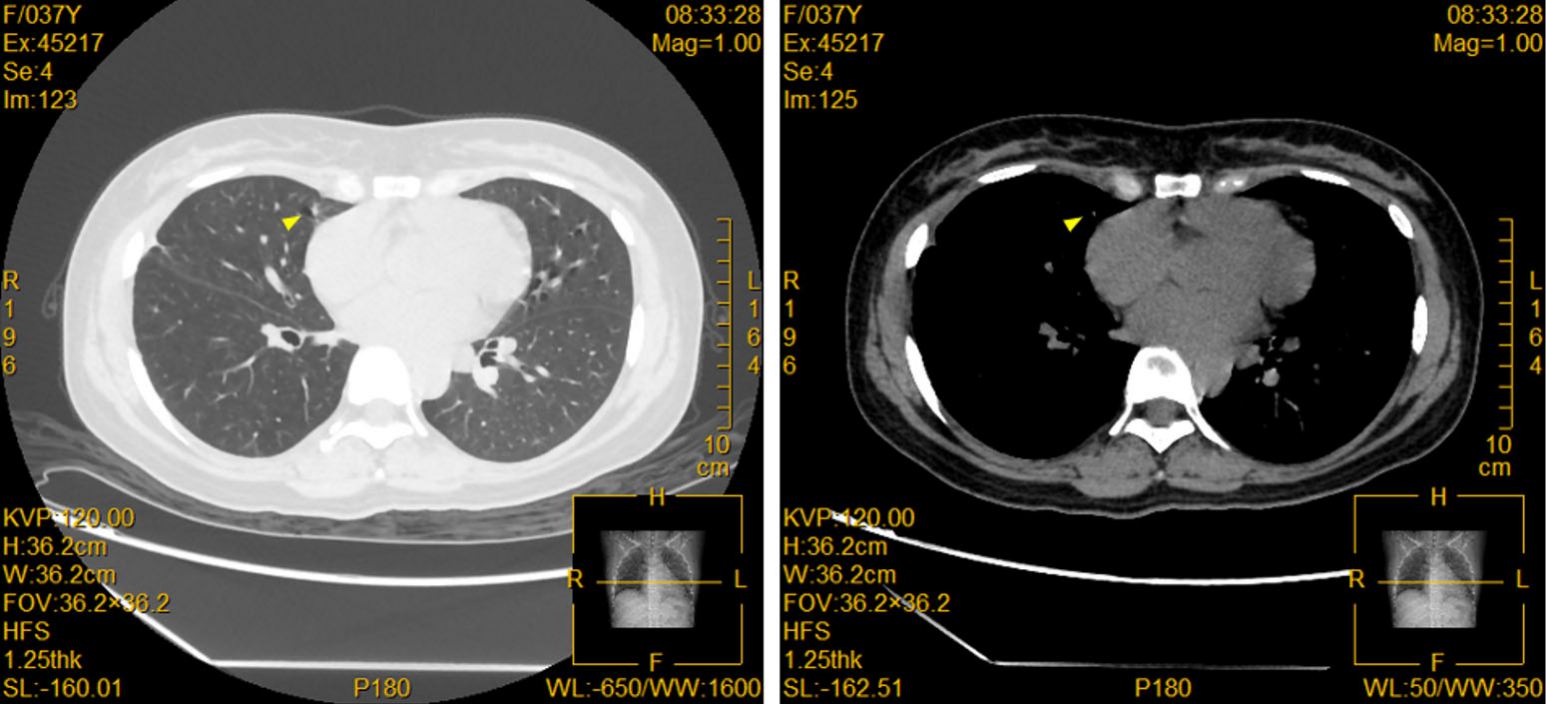
Enhanced CT shows significant shrinkage of the pulmonary lesion after treatment. A small patch of nodular shadow is seen in the middle lobe of the right lung, measuring approximately 7mm × 3mm in size, with some cord-like shadows and air cysts surrounding it (yellow triangle). A few scattered small nodules are seen in both lungs, some of which appear as ground-glass opacities. The largest of these is located in the posterior basal segment of the lower lobe of the right lung, measuring about 4mm in diameter, with slightly blurred edges.

Further testing with the human tumor 10-gene mutation panel revealed a positive result for the ALK fusion gene. On June 19, 2023, the patient began first-line anticancer treatment with oral 600mg Alectinib hydrochloride capsules twice daily. After two months of treatment, the results were surprisingly favorable: the ocular metastatic lesions completely regressed (**Figure 6**); the subpleural lesion in the right lower lung significantly absorbed, the solid nodule in the medial segment of the right middle lobe notably reduced in size, and the right hilar lymph nodes showed significant shrinkage, achieving partial remission (PR). By March 25, 2025, the right middle lobe nodule had shrunk from 16.6mm × 12.1mm to 7mm × 3mm (**Figure 7**).

**Figure 8:**
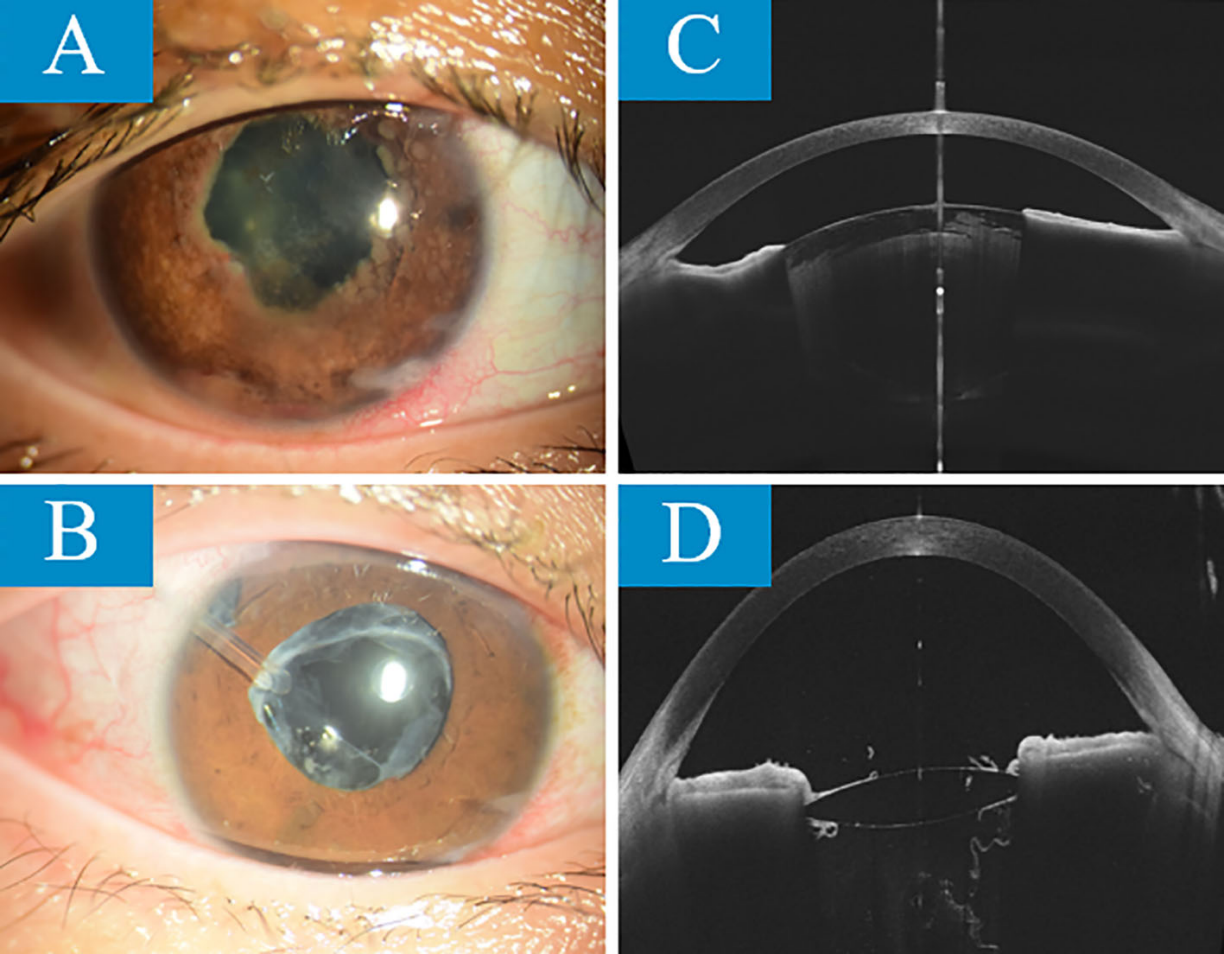
The comparison of anterior segment images taken with slit-lamp and OCT before targeted therapy **(A, C)** and 1 year and 5 months after treatment **(B, D)** shows the following. Before treatment, the patient’s iris texture was unclear, with posterior synechiae and multiple grayish-white nodules. The pupil was irregular, and the light reflex was absent. The lens was partially cloudy, and the remaining view under a small pupil was unclear. The anterior chamber was shallow. After treatment, the iris texture was still unclear, but the posterior synechiae were partially resolved. The grayish-white nodules had completely disappeared. The pupil’s light reflex was sluggish, the intraocular lens was in place, the drainage valve was in place, and the iris peripheral iridectomy was clearly visible. The anterior chamber depth had significantly increased.

In addition, the patient developed secondary glaucoma in the right eye with complete posterior iris adhesion. Despite the use of tobramycin-dexamethasone, pilocarpine, atropine sulfate, timolol eye drops, and oral acetazolamide, the intraocular pressure (IOP) remained consistently around 40mmHg. On May 10, 2023, the patient underwent right eye peripheral iridectomy, goniosynechialysis, and anterior chamber formation surgery, after which the IOP stabilized around 35mmHg. The patient also developed secondary cataracts in the right eye with a BCVA of 0.05. After the eye inflammation was relatively stable and after thorough communication with the patient, cataract phacoemulsification with intraocular lens implantation was performed on October 21, 2023. Post-surgery, the BCVA improved to 0.4, and the IOP was around 27mmHg. Due to poor IOP control, on January 28, 2024, the patient underwent right eye glaucoma valve implantation and anterior chamber formation surgery. Afterward, the IOP decreased to 18mmHg, and the BCVA recovered to 0.15. The patient was also prescribed non-steroidal and corticosteroid eye drops for inflammation, β-blockers, α_2_-adrenergic agonists, and prostaglandin F2α analogs for IOP reduction, as well as atropine sulfate gel for pupil dilation and prevention of iris-lens adhesions. The IOP stabilized at 18-20mmHg. On November 18, 2024, the patient developed significant posterior capsule opacification. After encouragement from the attending physician, posterior capsulotomy was performed, and the BCVA improved to 1.0. The BCVA has remained stable at 1.0 since then (comparative images of the anterior segment before and after targeted therapy are shown in **Figure 8**).

Overall, the patient exhibited good treatment tolerance. During the targeted therapy with Alectinib hydrochloride capsules, no obvious toxic effects were observed. The patient’s physical condition remained good, with no apparent immune toxicity or allergic reactions.

## 3 Discussion

This case report describes a 36-year-old female patient who initially presented with right eye anterior uveitis and secondary glaucoma, ultimately diagnosed with stage IV lung adenocarcinoma with intraocular and multi-system metastasis, a rare case. The clinical evolution, diagnostic challenges, and treatment response of this case provide important insights into the mechanisms of tumor metastasis and the clinical application of targeted therapy.

Ocular metastases account for 2-9% of all malignant tumors, with the majority originating from breast cancer and lung cancer, and 9-23% from pulmonary primary lesions (12). However, lung cancer metastasis to the eye presenting as anterior uveitis and secondary glaucoma is particularly rare and easily misdiagnosed as idiopathic inflammatory diseases. The uniqueness of this case lies in: 1) the patient is a young female with no smoking history, which does not fit the traditional high-risk group for lung cancer; 2) the patient presented with isolated anterior uveitis without typical respiratory symptoms. The patient initially exhibited typical signs of right eye anterior uveitis (corneal edema, KP (++), AR (+), cell (++), about 0.5mm hypopyon, irregular and dilated pupil (about 5mm), light reflex absent, complete posterior synechia, unclear iris texture, scattered grayish-white nodules on the surface) and significant intraocular pressure increase (43mmHg), with conventional anti-inflammatory and intraocular pressure-lowering treatments being ineffective, suggesting the need to consider non-inflammatory causes. A chest CT revealed a pulmonary primary lesion, which was confirmed as lung adenocarcinoma (TTF1+/CK7+) through bronchoscopy biopsy. Intraocular and central nervous system metastases were ultimately confirmed through aqueous humor cytology and brain MRI. This process highlights the importance of ocular symptoms as “sentinel manifestations” of systemic malignancies, especially in patients with treatment-resistant anterior uveitis, where tumor-related lesions should be actively ruled out. In the case of masquerade syndrome caused by ocular metastases, the high blood flow in the choroid makes it the most common site of metastasis (13). In contrast, the reduced blood flow in the anterior chamber makes anterior chamber metastases rarer, accounting for only 7-14% of intraocular metastases (14, 15). Notably, in rare cases, eye pain and symptoms resembling scleritis may indicate some potential metastases, and even serve as the initial manifestation of occult malignancies, with the mechanism related to tumor cells spreading hematogenously to the iris vasculature or the anterior ciliary arteries (16, 17).

The breakthrough in diagnosis for this case relied on multimodal imaging complementarity and integration of molecular pathology. Imaging-wise, the spiculated and lobulated features on chest CT (**Figure 1**) suggested malignancy, while the metabolic heterogeneity of the lesions seen on PET-CT (SUVmax 4.6-16.9) provided functional evidence for the localization of metastases. From the molecular pathology perspective, the tumor cells expressed TTF-1/CK7 and were negative for P40, which is characteristic of lung adenocarcinoma, while the detection of the ALK fusion gene directly changed the treatment strategy (18). ALK fusion occurs in about 5% of non-small cell lung cancer (NSCLC) cases and is most common in lung adenocarcinoma (19). Previous studies have shown that ALK-rearranged tumors are highly aggressive, prone to early metastasis, particularly to the central nervous system and eyes, at significantly higher rates than other subtypes (20, 21). In this case, besides ocular metastasis, the patient also had multiple brain parenchymal lesions (pons, cerebellum, frontal-parietal lobes). PET/CT further revealed involvement of mediastinal and axillary lymph nodes, which is consistent with the “multifocal metastasis” feature of ALK-positive tumors.

After 2 months of treatment with Alectinib hydrochloride capsules, the ocular lesions completely regressed (**Figure 6**) and the primary lesion shrank (PR), validating its excellent penetration to the central nervous system (22). Notably, the rapid relief of ocular metastasis after targeted therapy enabled subsequent vision reconstruction surgeries (cataract phacoemulsification + intraocular lens implantation, etc.) to achieve ideal results (BCVA 1.0). Continuous follow-up for 28 months showed the primary lesion stabilized at 7mm×3mm (**Figure 7**), far exceeding the median progression-free survival (PFS) of traditional chemotherapy (23). This provides a new paradigm for the long-term management of metastatic lung cancer.

The key to success in this case also lies in the timely selection of ophthalmic intervention: the patient underwent iris peripheral iridectomy + angle separation + anterior chamber formation surgery before systemic tumor control. Postoperatively, intraocular pressure was poorly controlled. However, after targeted drug therapy, cataract phacoemulsification + intraocular lens implantation and glaucoma valve implantation + anterior chamber formation were performed once the ocular inflammation stabilized. The patient’s ocular condition was effectively controlled after surgery, and no recurrence of ocular metastasis was observed. Furthermore, the patient’s adherence to treatment and regular follow-up visits exemplify the value of the doctor-patient relationship. This sequential treatment strategy allowed the patient to maintain functional vision while controlling the tumor, and the experience may be applicable to other patients with metastatic intraocular tumors.

## 4 Conclusion

This case emphasizes the need to include metastatic tumors in the differential diagnosis of treatment-resistant anterior uveitis. Combining multimodal imaging and fluid biopsies can improve the early diagnosis rate of intraocular metastases. Targeted drug use guided by human tumor 10 gene mutation combined detection highlights the importance of precision therapy. The timing of ophthalmic surgery should follow the principle of “systemic control first, functional reconstruction later.” Future research is needed to clarify the molecular mechanisms of intraocular metastases and the pharmacokinetic characteristics of targeted drugs in the eye to further optimize treatment strategies.

## Data Availability

The original contributions presented in the study are included in the article/supplementary material. Further inquiries can be directed to the corresponding author.
